# Survival Trends in Pediatric Differentiated Thyroid Cancer: A Middle Eastern Perspective

**DOI:** 10.3390/life14010158

**Published:** 2024-01-22

**Authors:** Akram Al-Ibraheem, Mohamed Al-Shammaa, Ahmed Saad Abdlkadir, Feras Istatieh, Ula Al-Rasheed, Thomas Pascual, Rawad Rihani, Hadeel Halalsheh, Taleb Ismael, Aysar Khalaf, Iyad Sultan, Issa Mohamad, Hikmat Abdel-Razeq, Asem Mansour

**Affiliations:** 1 Department of Nuclear Medicine and PET/CT, King Hussein Cancer Center (KHCC), Al-Jubeiha, Amman 11941, Jordan; 2 School of Medicine, University of Jordan, Amman 11942, Jordan; 3 Department of Nuclear Medicine, Radiotherapy and Nuclear Medicine Hospital, Bab Al-Muadham, Baghdad 10047, Iraq; 4 Department of Nuclear Medicine, Al-Amal National Hospital, Al-Andalus Square, Baghdad 10069, Iraq; 5 Philippine Nuclear Research Institute, Department of Science and Technology, Quezon City 1101, Philippines; 6 Department of Pediatrics, King Hussein Cancer Center (KHCC), Al-Jubeiha, Amman 11941, Jordan; 7 Department of Nuclear Medicine, Warith International Cancer Institute, Karbala 56001, Iraq; 8 Department of Radiation Oncology, King Hussein Cancer Center (KHCC), Al-Jubeiha, Amman 11941, Jordan; 9 Department of Medicine, King Hussein Cancer Center (KHCC), Al-Jubeiha, Amman 11941, Jordan; 10 Department of Diagnostic Radiology, King Hussein Cancer Center (KHCC), Al-Jubeiha, Amman 11941, Jordan

**Keywords:** pediatric thyroid cancer, Middle Eastern, event-free survival, children, distant metastasis, lymph node metastasis

## Abstract

Pediatric Differentiated Thyroid Cancer (pedDTC) is a rare pediatric malignancy with an increasing incidence over time. To date, there is a paucity of literature specifically addressing pedDTC within the context of Middle Eastern ethnicity. This retrospective study aimed to assess the risk-stratifying factors for overall survival (OS) and event-free survival (EFS) in pediatric DTC patients from Iraq and Jordan. The medical records of 81 patients from two tertiary cancer institutes were retrieved. Kaplan–Meier analysis was employed to investigate OS and EFS, and the Cox proportional hazards model was employed to estimate hazard ratios. All patients underwent surgery and radioactive iodine therapy, with a median age of 14 and an interquartile range of 12–15. Lymph node involvement was observed in 55% of cases, while distant metastases were present in 13.5%. After a median follow-up period of 68 months, the 10-year survival rate was determined to be 94%, while the 10-year EFS rate was 58%. EFS was negatively impacted by cervical lymph node metastases and early age of diagnosis (*p* ≤ 0.01, each). Therefore, pediatrics with initial cervical lymph node metastases and those diagnosed before puberty tend to experience poorer EFS, which may justify the need for more aggressive management plans.

## 1. Introduction

In recent decades, there has been an increasing prevalence of differentiated thyroid carcinoma (DTC) among the pediatric population [1]. In the pediatric population, DTC exhibits a heightened frequency of gene fusions, which impact the observed histological subcategories [2]. These gene fusions are also linked to more advanced extrathyroidal disease and provide distinct possibilities for targeted medical treatments. Moreover, molecular distinctions have been identified between pediatric and adult DTC, with a lower occurrence of BRAF mutations in pediatric cases and rare mutations in RAS genes [2,3,4]. These molecular discrepancies may contribute to the varied clinical behaviors observed in pediatric and adult DTC.

In regards to histopathologic subtypes, papillary thyroid carcinoma (PTC) is the predominant form of thyroid cancer found in children, making up approximately 90% of cases in the pediatric population [5]. Following this, follicular thyroid cancer is the second most common type, which is usually minimally invasive rather than widely invasive [2]. Therefore, children with a follicular subtype have a less aggressive disease than adults. In up to 70% of pediatric cases, extensive regional nodal involvement was noted, and 10–20% of patients had distant metastases [6]. The lungs are the most common site of distant metastasis [7]. Pediatric patients seem to have higher local and distant recurrence rates compared to adult patients, yet they typically exhibit prompt responses to treatment [8]. 

Fortunately, even when metastatic disease is present, long-term follow-up data indicate that children with DTC have long-term survival rates exceeding 90% [9,10,11]. It is noteworthy that even with distant metastases, children experience lower mortality rates compared to adults [12]. Furthermore, pulmonary metastases can persist without any significant progression for prolonged durations [13]. This positive prognosis can be attributed to the predominantly well-differentiated tumor types found in young patients, the infrequent occurrence of bone metastasis, and the favorable response of most tumors to radioactive iodine therapy [14].

Identifying specific risk factors for DTC in children is often challenging, as they are typically not discernible [14]. Nevertheless, a subset of patients does exhibit identifiable risk factors. Currently, the acknowledged risk factors for DTC in children include radiation exposure, genetic predisposition, and shared risk factors for developing a second primary neoplasm in cancer survivors. 

Radiation-induced thyroid cancer is exemplified by heightened rates observed in populations exposed to atomic blasts in Japan and fallout from above-ground nuclear weapon testing [15,16,17,18,19]. In the aftermath of the Chernobyl disaster, children or adolescents who were exposed to radiation during the fallout have demonstrated a notable rise in the occurrence of thyroid cancer [20]. Remarkably, the excess risk of developing thyroid cancer as a result of radiation exposure during childhood has endured for over five decades following the initial exposure [21]. Recent research also highlights the potential risk associated with routine medical procedures, as childhood dental X-rays have been linked to a twofold increase in DTC risk, indicating the complex influence of radiation on the development of DTC [22].

Notably, DTC is the most prevalent type of second malignancy observed in individuals who have been treated for Hodgkin’s and non-Hodgkin’s lymphomas, as well as the third most common neoplasm in leukemic children [23,24,25,26]. 

On the other hand, the genetic basis of DTC is emphasized by the occurrence of the disease within families and the presence of rare genetic syndromes. Familial nonmedullary differentiated thyroid cancer (FNMTC) is diagnosed when three or more family members are affected by DTC, indicating a hereditary inclination [27,28,29,30,31]. While current research efforts aim to identify specific genes associated with FNMTC, a comprehensive understanding of the molecular characteristics is still lacking [32,33]. Moreover, rare genetic syndromes like Cowden’s syndrome, which is caused by mutations in the Phosphatase and tensin homolog (PTEN) gene, emphasize the complex interaction between genetic factors and susceptibility to thyroid cancer [34,35].

As the role of ethnicity in disease manifestation and outcomes cannot be disregarded, it is imperative to investigate the patterns of disease presentation and survival within diverse ethnic groups in order to identify any variations or inconsistencies and appropriately address them. Consequently, the primary objective of this study was to analyze the patterns of disease presentation and survival in pediatric populations of Middle Eastern descent.

## 2. Materials and Methods

### 2.1. Data Collection

A retrospective examination was conducted to retrieve clinicopathological features of pediatrics diagnosed with Pediatric DTC (pedDTC) during the period from May 2005 to July 2021. The medical records of pediatric patients from two tertiary cancer centers were retrieved. These centers are based in Iraq and Jordan. All those patients were diagnosed with pedDTC below the age of 17 years. A case report form was used to collect the data set. Approval from Institutional Review Board (Study ID # 22 KHCC 123) was granted. The Iraqi participants were included in this study after the receipt of informed consent. In order to ensure the inclusion of those patients, written informed consent forms were obtained from either the patients themselves or their legal guardians. Adolescents who have the capacity to consent to publication can participate in giving consent along with their legal guardians [36]. Otherwise, for children and adolescents lacking the capacity to provide such consent, exclusive authorization was secured from their legal guardians [36]. The analysis excluded patients with undifferentiated histopathologic subtypes, as well as individuals with incomplete medical records that did not contain important pre-determined parameters. 

All enrolled patients underwent either a total or partial thyroidectomy. Patients who had a partial thyroidectomy and an established pedDTC diagnosis were subsequently offered a complete thyroidectomy. A cervical lymph node dissection was frequently performed along with this. The postoperative assessment encompassed a comprehensive examination to evaluate different clinicopathologic features. An analysis was conducted on variables such as age of diagnosis, gender, and histologic subtypes of the tumor. Additionally, the study examined primary tumor size and staging categories for the T, N, and M staging categories. The evaluation of TNM staging was carried out based on the classification guidelines provided by the 8th edition of the American Joint Committee on Cancer (AJCC) [37]. Furthermore, the risk of pedDTC recurrence was stratified based on the 2015 guidelines established by the American Thyroid Association (ATA) [38].

### 2.2. Postoperative Evaluation

Prior to the commencement of radioactive iodine therapy, cervical ultrasonography was performed on every patient. The evaluation of baseline levels of free thyroxine (T4), triiodothyronine (T3), thyroid-stimulating hormone (TSH), thyroglobulin (Tg), and thyroglobulin antibodies (TgAb) adhered to the guidelines established by the ATA.

The procedure of adjuvant radioactive iodine therapy was conducted between 4 and 6 weeks after the thyroid surgery in accordance with the protocols followed by the institution. The dosage administered ranged from 1.11 to 7.4 GBq. Patients who showed signs of local recurrence or distant metastases received additional radioactive iodine treatments.

### 2.3. Radioiodine Dosing

Dosing strategies for radioactive iodine encompassed both empirical fixed doses and personalized dosages based on either body surface area or weight [39]. Fixed doses comprised 1.11–3.7 GBq for exclusive primary pedDTC, 5.55 GBq for nodal involvement, and 5.55–7.4 GBq for distant metastases. In the case of children aged 10 years or younger, administered activities were adjusted based on body surface area or weight, employing the formula: pediatric dose = adult dose × body weight (kg)/70 kg [38]. Conversely, older children (>10 years) received fixed empirical doses [39,40]. To mitigate potential side effects, meticulous measures were implemented in strict accordance with the most recent ATA guidelines or earlier versions, depending on the timeframe of patient diagnosis. This approach aimed to achieve a well-balanced and patient-focused therapy [38].

### 2.4. Statistical Analysis

Initial descriptive statistics were calculated using appropriate statistical measures for continuous and categorical variables. The Kaplan–Meier method was employed to determine overall survival (OS) and event-free survival (EFS). OS was defined as the time from the initial diagnosis of the disease to any cause of death. EFS was defined as the time from the initial diagnosis of the disease to the progression or recurrence of the disease. The survival curves were compared using a log-rank test. Furthermore, the impact of prognostic factors on EFS was analyzed using a Cox proportional hazards model in both univariate and multivariate analyses. All statistical tests were conducted with a two-sided approach, and a significance level of *p* < 0.05 was utilized. The statistical software SPSS 27.0 for Windows (SPSS, Inc., Chicago, IL, USA) was utilized for data analysis. Subsequently, only factors that were found to be statistically significant are presented and analyzed.

## 3. Results

### 3.1. Patient and Disease Characteristics

A retrospective enrollment of 81 patients diagnosed with pedDTC was conducted. The median duration of follow-up for all pedDTC patients was found to be 68 months (interquartile range, 42–133 months). The female gender constituted the majority of the patients, accounting for 62% of the total. The median age at the time of diagnosis was 14, with an interquartile range from 12 to 15. Female predominance was noted with a 1.6 female/male ratio. The majority of patients (86%) were older than 10 years old, with only 14% being prepubertal (Table 1).

Histopathological examination unveiled PTC predominance. The median tumor size is 3.3 cm (interquartile range, 2.5–3.7 cm). Histopathological examination revealed the presence of cervical lymph node metastases in 45 patients (63% with N1a and 37% with N1b). Distant metastasis was observed in 13.5% of cases. Lung metastasis was documented in all these cases (Table 2).

It is noteworthy that all patients have undergone surgery and radioactive iodine therapy. The main aim of this approach was to eliminate pedDTC and achieve complete remission. In about one-third of patients, multiple doses of radioactive iodine were necessary. Sixteen patients were offered two doses of radioactive iodine, seven received three doses, and three patients were exposed to an excess of three doses, specifically four or five. The remaining two-thirds achieved satisfactory responses with only one dose of radioactive iodine therapy (Table 3).

### 3.2. Survival Outcome

**Figure 1:**
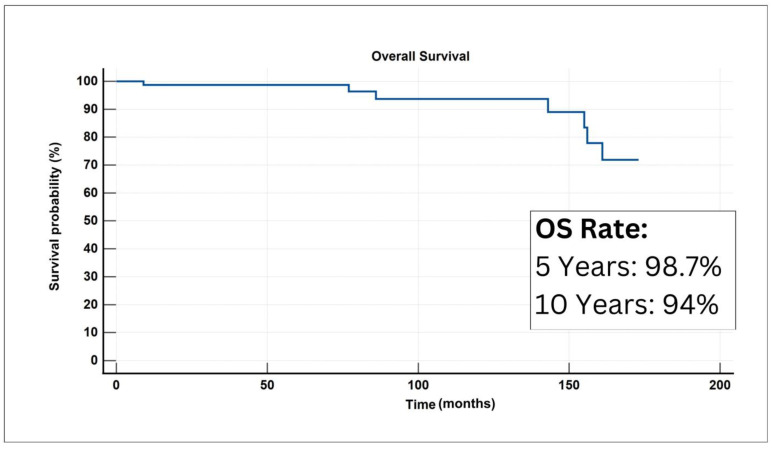
A graphical plot for the Kaplan–Meir curve denoting overall survival analysis for the study cohort.

Out of the total of 81 cases, only seven deaths from pedDTC were recorded during the median follow-up duration of 68 months. Notably, there were four fatalities documented as a result of pulmonary metastasis, whereas three other fatalities were ascribed to lung, thoracic lymph nodes, and bone metastases. All deceased patients were offered multiple radioactive iodine doses (ranging from 3–5). Disease events were recorded in 28 cases. The majority of these events were related to disease recurrence (n = 14). To a lesser extent, disease progression and lung metastases were observed (n = 10 and n = 4, respectively). The 5-year and 10-year OS were found to be 98.7% and 94%, respectively (Figure 1). 

**Figure 2:**
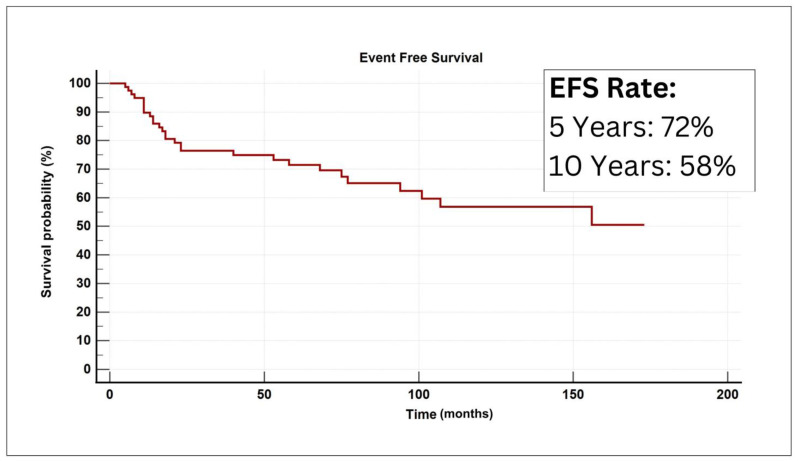
A graphical plot for the Kaplan–Meir curve denoting event-free survival analysis for the study cohort.

Meanwhile, the 5-year and 10-year EFS were 72% and 58%, respectively (Figure 2).

Cox proportional hazard analysis was implemented to examine the influence of various disease-related factors on EFS. These include age at diagnosis, gender, family history of thyroid malignancy, nodal disease, distant metastatic disease, ATA risk of recurrence, overall staging, and histopathologic subtype. On univariate Cox proportional hazard analysis, EFS was negatively influenced by younger age at diagnosis, nodal metastases, and distant metastases (Table 4). 

Among these, only nodal metastases and early age of diagnosis were retained as significant negative EFS influencers on multivariate analysis (Table 5).

## 4. Discussion

As the incidence of pedDTC increases worldwide, the significance of evidence-based guidelines at a national level becomes crucial in enabling healthcare providers to offer consistent and customized care [41]. This focuses on individualized treatment, considering both patient-specific attributes and disease-related factors, which is a fundamental aspect of current medical practice. Unfortunately, there is a notable lack of comprehensive and reliable evidence for cases of pedDTC. Professional organizations like the ATA and the British Society for Pediatric Endocrinology and Diabetes have released guidelines to provide consensus [38,42]. However, these recommendations are largely based on available data sources, which mainly consist of retrospective single-center case series and expert opinions. This reliance on limited data sources highlights the difficulties faced in managing pedDTC and emphasizes the need to expand the evidence base through dedicated efforts. It is important to note that there are distinct clinical characteristics of DTC in children compared to adults. Specifically, pedDTC often presents at more advanced stages, but it responds well to treatment and has a generally positive prognosis [8]. However, there is a lack of observational studies specific to children of Middle Eastern, South American, and South African ethnicities. Since the presentation and outcomes of DTC can vary among different ethnic groups, it is crucial to conduct ethnically diverse research to fill these knowledge gaps. This will allow for a better understanding of the nuances of pedDTC in different demographic and clinical contexts and provide optimal care for patients. Taking this into consideration, we commenced a comprehensive data-gathering endeavor on children and adolescents from Iraq and Jordan who exhibited pedDTC. Through this extensive study, we have ascertained that the prognosis for this condition is favorable, as evidenced by the relatively low number of fatalities. To the best of our knowledge, our study is one of the largest multicentric studies to examine the survival trends in pediatrics with Middle Eastern ethnicity. In our cohort, pediatrics had shown to have a high incidence of metastatic disease (55% nodal metastasis and 14% distant metastasis). When compared to the adult Middle Eastern population of the same ethnicity, pediatrics have a much higher rate of disease events [43]. Despite the incidence of seven documented deaths, our analysis shows that the OS rate among pedDTC patients is still highly favorable.

Upon retrospectively examining the survival patterns of various ethnic groups, it becomes evident that all groups exhibited positive outcomes. To the best of our current understanding, the majority of research studies on pedDTC patients have predominantly focused on individuals of European descent. In the United Kingdom, a comprehensive nationwide observational study was conducted to evaluate survival trends [41]. The results of this study indicated that despite its aggressive initial manifestation, pedDTC demonstrated favorable long-term outcomes [41]. In Romania, a long-term analysis was conducted on 62 patients to assess survival outcomes, revealing an excellent prognosis with an overall survival rate of 100% [44]. Similarly, a single-centric Indian cohort also exhibited favorable survival trends [45]. Danese et al. conducted a retrospective study in Italy with the objective of determining the prevalence, clinical presentation, and outcome of thyroid carcinoma in the pediatric and adolescent population [46]. Data from 48 patients were obtained, all of whom had no prior history of head and neck irradiation [46]. Following an average monitoring period of 58.4 months, it was found that 50% of the patients had cervical lymph node metastases, while 4.2% had lung metastases [46]. Mihailovic and his colleagues undertook a study in Serbia to investigate the predictive factors for patients with DTC [47]. The study included a total of 51 patients, who were further divided into three age groups: children, adolescents, and adults [47]. It is worth noting that nearly half of the participants were under the age of 16 [47]. The results revealed that the probabilities of recurrence were 16.7% at the 5-year mark, 22.3% at the 10-year mark, and 33.3% at both the 15-year and 23-year marks following the initial treatment [47]. Age, initial treatment, and tumor multifocality were found to significantly impact the likelihood of recurrence [47]. In another separate investigation conducted in Belarus, Demidchik et al. examined a cohort of 740 children diagnosed with DTC [48]. Their findings revealed a significant correlation between the likelihood of DTC recurrence and various factors, including tumor stage, the extent of surgical intervention, the age of the pediatric patient, and the manifestation of symptoms at the time of diagnosis [48]. In a study conducted by Enomoto et al. (2016), a total of 142 pediatric patients diagnosed with DTC were examined over a median follow-up period of 22 years [49]. The findings indicated that individuals under the age of 16 were identified as a susceptible group for recurrent disease [49]. Additionally, other significant risk factors for recurrent DTC were identified, including a family history of thyroid cancer, tumor size, extensive lymph node metastases, invasion into surrounding tissues, and initial distant metastases [49]. In a survey conducted through questionnaires, data was gathered from 114 children and adolescents diagnosed with DTC from 65 clinical institutions in Germany [50]. The study examined the characteristics of 80 females and 34 males and investigated the impact of age, gender, histology, multicentric growth, tumor stage, and lymph node involvement on the occurrence of distant metastases [50]. The findings revealed a notable increase in the incidence of PTC in females, particularly during puberty [50]. It was also observed that childhood thyroid carcinoma often presents with lymph node involvement, distant metastases, and infiltration of tumors outside the thyroid gland [50]. Additionally, the study identified that the advanced local–regional extension stage is a significant factor influencing the likelihood of distant metastases in children [50]. In a study conducted in Poland, Jarzab et al. examined a cohort of 102 children diagnosed with DTC who were monitored for an average duration of 5 years [51]. Throughout the entire follow-up period, no deaths attributable to cancer were recorded [51]. The findings of this investigation exhibited a comparable trend in EFS to our own research outcomes [51]. Zbitou and colleagues conducted a study in France on individuals with pedDTC, analyzing a population-based sample of 774 cases of thyroid cancer [52]. The predominant histopathologic subtype observed in the majority of patients was PTC [52]. pedDTC was found to be more prevalent in females and adolescents [52]. Thyroidectomy was performed on almost all patients, and in most cases, it was accompanied by the administration of adjuvant radioactive iodine therapy [52]. The median follow-up period was 11.3 years for children and 5.7 years for adolescents, during which the 5-year survival rate exceeded 98.5% [52]. Despite the abundance of observational studies on DTC survival trends, many of these studies fail to account for pediatric patients as a distinct group with their own survival outcomes and include patients aged below 20 years of age.

To a lesser extent, a smaller proportion of studies have also examined pediatric patients from the United States and East Asia. Seeking to provide updated insights into survival patterns within the United States, Zhang et al. conducted a study utilizing data from the Surveillance, Epidemiology, and End Results (SEER) program [53]. This investigation revealed a notably high cumulative survival rate for childhood thyroid cancer, ranging from 97 to 99% [53]. In an attempt to compare the survival outcomes of individuals with pedDTC following nuclear accidents in Belarus (post-Chernobyl) and Japan (post-Fukushima), Drodz et al. conducted a review of relevant studies [54]. The analysis demonstrated that the long-term overall survival of children with pedDTC from Belarus affected by the Chernobyl incident was remarkably good, with a 97% survival rate after a duration of 15 years [54]. For Japanese, the rates of disease-free survival after 10, 20, and 30 years were found to be 83.8%, 71.7%, and 53.5%, respectively [54]. An additional study conducted by Wada et al. in Japan investigated the outcomes and risk factors in children with DTC who were classified as TNM stage I [55]. The study found that male gender, tumor stage, and lymphadenopathy can have a negative impact on event-free survival in stage I pedDTC patients [55]. More recently, Harahap et al. conducted a retrospective study at a single center in Indonesia to examine the clinicopathologic factors and survival outcomes of pedDTC patients [56]. Notably, cervical lymph node involvement was observed in nearly one-fourth of the study sample, while distant metastasis occurred in one-fifth of pedDTC patients [56]. The majority of patients had stage I pedDTC [56]. Disease recurrence was observed in one-third of patients over a median follow-up period of 2 years [56]. In China, a retrospective study conducted by Lee et al. investigated the clinicopathologic characteristics and outcomes of 34 pediatric patients with DTC over a span of 35 years [57]. The majority of patients received adjuvant radioactive iodine treatment [57]. The presence of cervical lymph node metastases can raise the probability of disease recurrence [57]. A single patient who had an advanced locoregional disease experienced multiple rounds of disease recurrence followed by anaplastic transformation [57]. Unfortunately, this patient died 18 years after the initial pedDTC diagnosis [57]. However, it was generally concluded that pedDTC has an indolent nature as death is a rare occurrence [57]. Nevertheless, frequent recurrence of pedDTC necessitates careful and diligent follow-up for this challenging age group. Huang et al. conducted a study in Taiwan that yielded similar positive OS results [58]. The researchers retrospectively analyzed data from 116 pediatric patients diagnosed with DTC over an average follow-up period of 11 years [58]. In comparison to adult patients with DTC, the younger patients with papillary and follicular subtypes had lower EFS rates [58]. However, the cancer survival rates were higher in the younger age group compared to patients aged 45 years and above [58].

At the time of initial diagnosis, patients with pedDTC exhibit more advanced disease compared to adults [59,60,61,62]. Additionally, children often experience lung metastases, which persist even after multiple radioactive iodine therapies [63,64]. However, death is uncommon and typically occurs many years after diagnosis [65]. The long-term survival rate for children with DTC is approximately 90% at 30 years post-diagnosis [9,59,60,61,62]. Nevertheless, pediatric patients have a higher likelihood of cancer recurrence, especially those with multifocal papillary thyroid cancer, significant tumor extension beyond the thyroid capsule, and extensive lymph node involvement [8]. Pediatric patients with pretherapeutic Tg levels exceeding 10 ng/mL are at a higher risk of persistent structural disease, particularly those classified as intermediate risk according to the ATA guidelines [38,66,67].

The primary focus of debate among healthcare professionals regarding pedDTC patients revolves around treatment decisions and planning. The recommended treatment strategy typically involves total thyroidectomy followed by radioactive iodine therapy [55]. However, it is important to note that total thyroidectomy is associated with a higher risk of surgical complications, particularly in pediatric patients. Aggressive surgery, such as total thyroidectomy, has been linked to significant complications, including permanent hypoparathyroidism and recurrent laryngeal nerve palsy in a considerable percentage of pedDTC patients [68]. Nevertheless, it is advisable to consider implementing a total thyroidectomy performed by skilled surgeons as a means to reduce the likelihood of severe complications and achieve optimal results [69]. Such an approach was further supported by Dottorini et al.’s investigation in Italy, which involved a comprehensive analysis of 85 patients with pedDTC over a span of 37 years [70]. The study demonstrated that radioactive iodine therapy is remarkably efficacious in diminishing lung metastases, although attaining undetectable levels of Tg is infrequently observed [70]. Consequently, total thyroidectomy and radioactive iodine therapy emerge as effective and safe treatment modalities for the majority of pedDTC patients [70]. This has been endorsed lately by novel interdisciplinary guidelines from the European Thyroid Association [71].

Ethnicity has been found to have an impact on the presentation and outcomes of pedDTC. Previous research has identified distinct patterns in disease presentation and outcomes among different ethnic groups. This influence was initially observed in the United States, where there is a multiracial population. Specifically, individuals of non-White and Hispanic backgrounds tend to present with larger tumors and more advanced stages of pediatric differentiated thyroid cancer [72]. Another study showed a higher incidence of lymph node involvement in Hispanic pediatric patients [73]. Furthermore, a disparity in OS rates was documented, with Black patients having worse OS compared to White and Hispanic patients [73]. Disparities in OS and EFS of pedDTC have been observed among different ethnicities in terms of overall survival and event-free survival rates at different time points [10]. In contrast to the majority of studies conducted in the United States and Europe, our study revealed a lower overall survival rate and a higher incidence of disease-related events. Consequently, it is imperative to conduct future large-scale studies on the Middle Eastern population with pediatric differentiated thyroid carcinoma in order to investigate the significant factors that contribute to such evidence. 

The study was limited by its retrospective nature, with data lacking on some histopathologic and genetic characteristics. However, it remains the first and only multicentric study to observe survival trends in Middle Eastern pediatrics.

## 5. Conclusions

Our research, which involved the largest multicenter group of pedDTC in the Middle Eastern population, showed generally favorable OS rates. Children who have initial lymph node metastases and are diagnosed before reaching puberty have a higher propensity for experiencing a greater number of disease events. Consequently, it is advisable to employ a more proactive strategy in the management and surveillance of these patients, with the aim of mitigating the occurrence of disease events.

## Figures and Tables

**Table 1:** Demographics of the study sample.

Demographics	
**Age At Diagnosis (in Years)**	
Median	14 years
Interquartile Range	12–15 years
**Gender (Number, Percentage)**	
Female	50, (62%)
Male	31, (39%)
**Nationality (Number, Percentage)**	
Iraq	51, (63%)
Jordan	30, (37%)

**Table 2:** Histopathological characteristics of the study sample.

**Histopathological Characteristics**	
**Histologic Subtypes (Number, Percentage)**	
Papillary	74, (91.3%)
Follicular	6, (7.4%)
Hurthle	1, (1.3%)
**Tumor Size (cm median and interquartile range)**	3.3, (2.5–3.7)
**T-Category (Number, Percentage)**	
T1b	10, (12.3%)
T2	44, (54.3%)
T3a	8, (9.9%)
T3b	9, (11.1%)
T4a	6, (7.4%)
T4b	4, (5%)
**N-Category (Number, Percentage)**	
N0	36, (44.5%)
N1a	27, (33.3%)
N1b	18, (22.2%)
**M-Category (Number, Percentage)**	
M0	70, (86.4%)
M1	11, (13.5%)
**Overall Staging (Number, Percentage), according to AJCC ^1^ 8th edition**	
Stage I	70, (86.4%)
Stage II	11, (13.5%)

^1^ AJCC: American Joint Committee on Cancer.

**Table 3:** Summary table for the various therapy modalities and risk stratification applied to the study sample.

Treatment Modality (Number, Percentage)	
Total Thyroidectomy	68, (84%)
Partial Thyroidectomy	13, (16%)
Completion thyroidectomy	13, (16%)
RAI ^1^	81, (100%)
**RAI Treatment Received (Number, Percentage)**	
Single Dose	55, (68%)
Multiple Doses	26, (32%)
**Cumulative RAI dose (GBq median and interquartile range)**	3.7, (2.5–7.4)
**Risk of Recurrence (Number, Percentage), according to 2015 ATA ^2^ guidelines**	
Low Risk	36, (44.4%)
Intermediate Risk	21, (26%)
High Risk	24, (29.6%)

^1^ RAI: Radioactive Iodine; ^2^ ATA: American Thyroid Association.

**Table 4:** Table illustrating Cox Proportional Hazard univariate analysis for the significance of various factors on Event-free Survival.

Univariate Analysis			
Variable	Hazard Ratio	95% Confidence Interval	*p*-Value
Age At Diagnosis (Below 10 years of age vs. >10 years)	0.4	0.2–0.9	0.005
Nodal Metastases	4.2	1.5–10.8	0.003
Distant Metastases	0.3	0.2–0.7	0.02

**Table 5:** Table illustrating Cox Proportional Hazard multivariate analysis of the significance of various factors on Event-free Survival.

Multivariate Analysis			
Variable	Hazard Ratio	95% Confidence Interval	*p*-Value
Age At Diagnosis (Below 10 years of age vs. >10 years)	0.4	0.2–0.9	0.03
Nodal Metastases	4.5	1.4–14.7	0.01

## Data Availability

The data provided in this research can be obtained upon reasonable request.

## References

[1] Bernier M.O., Withrow D.R., Berrington de Gonzalez A., Lam C.J., Linet M.S., Kitahara C.M., Shiels M.S. (2019). Trends in pediatric thyroid cancer incidence in the United States, 1998–2013. Cancer.

[2] Paulson V.A., Rudzinski E.R., Hawkins D.S. (2019). Thyroid cancer in the pediatric population. Genes.

[3] Gertz R.J., Nikiforov Y., Rehrauer W., McDaniel L., Lloyd R.V. (2016). Mutation in BRAF and other members of the MAPK pathway in papillary thyroid carcinoma in the pediatric population. Arch. Pathol. Lab. Med..

[4] Pekova B., Sykorova V., Dvorakova S., Vaclavikova E., Moravcova J., Katra R., Astl J., Vlcek P., Kodetova D., Vcelak J. (2020). RET, NTRK, ALK, BRAF, and MET fusions in a large cohort of pediatric papillary thyroid carcinomas. Thyroid.

[5] Dermody S., Walls A., Harley Jr E.H. (2016). Pediatric thyroid cancer: An update from the SEER database 2007–2012. Int. J. Pediatr. Otorhinolaryngol..

[6] Liu Z., Hu D., Huang Y., Chen S., Zeng W., Zhou L., Zhou W., Wang M., Feng H., Wei W. (2019). Factors associated with distant metastasis in pediatric thyroid cancer: Evaluation of the SEER database. Endocr. Connect..

[7] Ngo D.Q., Tran T.D., Ngo Q.X., Van Le Q. (2020). Miliary pulmonary metastases from well-differentiated pediatric thyroid carcinoma. J. Pediatr. Surg. Case Rep..

[8] Lee Y.A., Jung H.W., Kim H.Y., Choi H., Kim H.-Y., Hah J.H., Park D.J., Chung J.-K., Yang S.W., Shin C.H. (2015). Pediatric patients with multifocal papillary thyroid cancer have higher recurrence rates than adult patients: A retrospective analysis of a large pediatric thyroid cancer cohort over 33 years. J. Clin. Endocrinol. Metab..

[9] O’Gorman C.S., Hamilton J., Rachmiel M., Gupta A., Ngan B.Y., Daneman D. (2010). Thyroid cancer in childhood: A retrospective review of childhood course. Thyroid.

[10] Rachmiel M., Charron Μ., Gupta A., Hamilton J., Wherrett D., Forte V., Daneman D. (2006). Evidence-based review of treatment and follow up of pediatric patients with differentiated thyroid carcinoma. J. Pediatr. Endocrinol. Metab..

[11] Powers P.A., Dinauer C.A., Tuttle R.M., Robie D.K., McClellan D.R., Francis G.L. (2003). Tumor size and extent of disease at diagnosis predict the response to initial therapy for papillary thyroid carcinoma in children and adolescents. J. Pediatr. Endocrinol. Metab..

[12] Brink J.S., Van Heerden J.A., McIver B., Salomao D.R., Farley D.R., Grant C.S., Thompson G.B., Zimmerman D., Hay I.D. (2000). Papillary thyroid cancer with pulmonary metastases in children: Long-term prognosis. Surgery.

[13] La Quaglia M.P., Black T., Holcomb G.W., Sklar C., Azizkhan R.G., Haase G.M., Newman K.D. (2000). Differentiated thyroid cancer: Clinical characteristics, treatment, and outcome in patients under 21 years of age who present with distant metastases. A report from the Surgical Discipline Committee of the Children’s Cancer Group. J. Pediatr. Surg..

[14] Rivkees S.A., Mazzaferri E.L., Verburg F.A., Reiners C., Luster M., Breuer C.K., Dinauer C.A., Udelsman R. (2011). The treatment of differentiated thyroid cancer in children: Emphasis on surgical approach and radioactive iodine therapy. Endocr. Rev..

[15] Preston D., Ron E., Tokuoka S., Funamoto S., Nishi N., Soda M., Mabuchi K., Kodama K. (2007). Solid cancer incidence in atomic bomb survivors: 1958–1998. Radiat. Res..

[16] Watanabe S., Shimosato Y., Ohkita T., Ezaki H., Shigemitsu T., Kamata N. (1972). Leukemia and thyroid carcinoma found among A-bomb survivors in Hiroshima. Current Problems in the Epidemiology of Cancer and Lymphomas.

[17] Davis S., Kopecky K.J., Hamilton T.E., Onstad L., Team H.T.D.S. (2004). Thyroid neoplasia, autoimmune thyroiditis, and hypothyroidism in persons exposed to iodine 131 from the Hanford nuclear site. JAMA.

[18] Robbins J., Schneider A.B. (2000). Thyroid cancer following exposure to radioactive iodine. Rev. Endocr. Metab. Disord..

[19] Gilbert E.S., Huang L., Bouville A., Berg C.D., Ron E. (2010). Thyroid cancer rates and 131I doses from Nevada atmospheric nuclear bomb tests: An update. Radiat. Res..

[20] Brenner A.V., Tronko M.D., Hatch M., Bogdanova T.I., Oliynik V.A., Lubin J.H., Zablotska L.B., Tereschenko V.P., McConnell R.J., Zamotaeva G.A. (2011). I-131 dose response for incident thyroid cancers in Ukraine related to the Chornobyl accident. Environ. Health Perspect..

[21] Furukawa K., Preston D., Funamoto S., Yonehara S., Ito M., Tokuoka S., Sugiyama H., Soda M., Ozasa K., Mabuchi K. (2013). Long-term trend of thyroid cancer risk among Japanese atomic-bomb survivors: 60 years after exposure. Int. J. Cancer.

[22] Memon A., Godward S., Williams D., Siddique I., Al-Saleh K. (2010). Dental x-rays and the risk of thyroid cancer: A case-control study. Acta Oncol..

[23] Sankila R., Garwicz S., Olsen J., Döllner H., Hertz H., Kreuger A., Langmark F., Lanning M., Möller T., Tulinius H. (1996). Risk of subsequent malignant neoplasms among 1,641 Hodgkin’s disease patients diagnosed in childhood and adolescence: A population-based cohort study in the five Nordic countries. Association of the Nordic Cancer Registries and the Nordic Society of Pediatric Hematology and Oncology. J. Clin. Oncol..

[24] Davies S.M. (2007). Subsequent malignant neoplasms in survivors of childhood cancer: Childhood Cancer Survivor Study (CCSS) studies. Pediatr. Blood Cancer.

[25] Maule M., Scélo G., Pastore G., Brennan P., Hemminki K., Pukkala E., Weiderpass E., Olsen J., Tracey E., McBride M. (2008). Risk of second malignant neoplasms after childhood central nervous system malignant tumours: An international study. Eur. J. Cancer.

[26] Tucker M., Jones P.M., Boice Jr J., Robison L., Stone B., Stovall M., Jenkin R., Lubin J., Baum E., Siegel S. (1991). Therapeutic radiation at a young age is linked to secondary thyroid cancer. Cancer Res..

[27] Malchoff C.D., Malchoff D.M. (2005). Familial papillary thyroid carcinoma. Molecular Basis of Thyroid Cancer.

[28] Ozaki O., Ito K., Kobayashi K., Suzuki A., Manabe Y., Hosoda Y. (1988). Familial occurrence of differentiated, nonmedullary thyroid carcinoma. World J. Surg..

[29] Körber C., Geling M., Werner E., Mörtl M., Mäder U., Reiners C., Farahati J. (2000). Incidence of familial non-medullary thyroid carcinoma in the patient register of the Clinic and Polyclinic of Nuclear Medicine, University of Würzburg. Nuklearmedizin. Nucl. Med..

[30] Hillenbrand A., Varhaug J.-E., Brauckhoff M., Pandev R., Haufe S., Dotzenrath C., Köberle R., Hoffmann R., Klein G., Kadmon M. (2010). Familial nonmedullary thyroid carcinoma—Clinical relevance and prognosis. A European multicenter study: ESES Vienna presentation. Langenbeck’s Arch. Surg..

[31] Parameswaran R., Brooks S., Sadler G.P. (2010). Molecular pathogenesis of follicular cell derived thyroid cancers. Int. J. Surg..

[32] Morrison P.J., Atkinson A.B. (2009). Genetic aspects of familial thyroid cancer. Oncologist.

[33] Tran T., Gianoukakis A.G. (2010). Familial thyroid neoplasia: Impact of technological advances on detection and monitoring. Curr. Opin. Endocrinol. Diabetes Obes..

[34] Blumenthal G.M., Dennis P.A. (2008). PTEN hamartoma tumor syndromes. Eur. J. Hum. Genet..

[35] Farooq A., Walker L., Bowling J., Audisio R. (2010). Cowden syndrome. Cancer Treat. Rev..

[36] Mathews B. (2022). Adolescent Capacity to Consent to Participate in Research: A Review and Analysis Informed by Law, Human Rights, Ethics, and Developmental Science. Laws.

[37] Amin M.B., Edge S.B., Greene F.L., Byrd D.R., Brookland R.K., Washington M.K., Gershenwald J.E., Compton C.C., Hess K.R., Sullivan D.C. (2017). AJCC Cancer Staging Manual.

[38] Francis G.L., Waguespack S.G., Bauer A.J., Angelos P., Benvenga S., Cerutti J.M., Dinauer C.A., Hamilton J., Hay I.D., Luster M. (2015). Management guidelines for children with thyroid nodules and differentiated thyroid cancer: The American Thyroid Association guidelines task force on pediatric thyroid cancer. Thyroid.

[39] Tamam M., Uyanik E., Edís N., Mulazimoglu M., Ozpacaci T. (2020). Differentiated thyroid carcinoma in children: Clinical characteristics and long-term follow-up. World J. Nucl. Med..

[40] Luster M., Clarke S., Dietlein M., Lassmann M., Lind P., Oyen W., Tennvall J., Bombardieri E. (2008). Guidelines for radioiodine therapy of differentiated thyroid cancer. Eur. J. Nucl. Med. Mol. Imaging.

[41] Lee K., Sharabiani M., Tumino D., Wadsley J., Gill V., Gerrard G., Sindhu R., Gaze M., Moss L., Newbold K. (2019). Differentiated thyroid cancer in children: A UK multicentre review and review of the literature. Clin. Oncol..

[42] Spoudeas H., Albanese A., Saran F. (2005). Paediatric Endocrine Tumours: A Multi-Disciplinary Consensus Statement of Best Practice from a Working Group Convened Under the Auspices of the The British Society of Paediatric Endocrinology & Diabetes and the United Kingdom Children’s Cancer Study Group.

[43] Al-Ibraheem A., Al-Rasheed U., Mashhadani N., Abdlkadir A.S., Al-Adhami D.A., Ruzzeh S., Istatieh F., Mansour A., Hamdan B., Kheetan R. (2023). Long-Term Survival Analysis and Prognostic Factors of Arabic Patients with Differentiated Thyroid Carcinoma: A 20-Year Observational Study at the King Hussein Cancer Center (KHCC) Involving 528 Patients. Cancers.

[44] Ștefan A.-I., Piciu A., Căinap S.S., Gabora K., Piciu D. (2020). Differentiated thyroid cancer in children in the last 20 years: A regional study in Romania. J. Clin. Med..

[45] Thankamony P., Nirmal G., Chandar R., Nair A.K., Veeramoni Iyer Mriduladevi P. (2021). Differentiated thyroid carcinoma in children: A retrospective analysis of 125 pediatric cases from a single institution in India. Pediatr. Blood Cancer.

[46] Danese D., Gardini A., Farsetti A., Sciacchitano S., Andreoli M., Pontecorvi A. (1997). Thyroid carcinoma in children and adolescents. Eur. J. Pediatr..

[47] Mihailovic J., Nikoletic K., Srbovan D. (2014). Recurrent disease in juvenile differentiated thyroid carcinoma: Prognostic factors, treatments, and outcomes. J. Nucl. Med..

[48] Demidchik Y.E., Demidchik E.P., Reiners C., Biko J., Mine M., Saenko V.A., Yamashita S. (2006). Comprehensive clinical assessment of 740 cases of surgically treated thyroid cancer in children of Belarus. Ann. Surg..

[49] Enomoto Y., Enomoto K., Uchino S., Shibuya H., Watanabe S., Noguchi S. (2012). Clinical features, treatment, and long-term outcome of papillary thyroid cancer in children and adolescents without radiation exposure. World J. Surg..

[50] Farahati J., Bucsky P., Parlowsky T., Mäder U., Reiners C. (1997). Characteristics of differentiated thyroid carcinoma in children and adolescents with respect to age, gender, and histology. Cancer Interdiscip. Int. J. Am. Cancer Soc..

[51] Jarząb B., Junak D.H., Włoch J., Kalemba B., Roskosz J., Kukulska A., Puch Z. (2000). Multivariate analysis of prognostic factors for differentiated thyroid carcinoma in children. Eur. J. Nucl. Med..

[52] Zbitou A., Desandes E., Guissou S., Mallebranche C., Lacour B. (2022). Thyroid cancers in children and adolescents in France: Incidence, survival and clinical management over the 2000–2018 period. Int. J. Pediatr. Otorhinolaryngol..

[53] Zhang B., Wu W., Shang X., Huang D., Liu M., Zong L. (2022). Incidence and prognosis of thyroid cancer in children: Based on the SEER database. Pediatr. Surg. Int..

[54] Drozd V., Saenko V., Branovan D.I., Brown K., Yamashita S., Reiners C. (2021). A search for causes of rising incidence of differentiated thyroid cancer in children and adolescents after Chernobyl and Fukushima: Comparison of the clinical features and their relevance for treatment and prognosis. Int. J. Environ. Res. Public Health.

[55] Wada N., Sugino K., Mimura T., Nagahama M., Kitagawa W., Shibuya H., Ohkuwa K., Nakayama H., Hirakawa S., Rino Y. (2009). Pediatric differentiated thyroid carcinoma in stage I: Risk factor analysis for disease free survival. BMC Cancer.

[56] Harahap A.S., Sari D.G., Stephanie M., Siswoyo A.D., Zaid L.S.M., Kartini D., Ham M.F., Tarigan T.J.E. (2022). Clinicopathological Profile of Thyroid Carcinoma in Young Patients: An Indonesian Single-Center Study. J. Thyroid Res..

[57] Lee Y.-M., Lo C.-Y., Lam K.-Y., Wan K.-Y., Tam P.K. (2002). Well-differentiated thyroid carcinoma in Hong Kong Chinese patients under 21 years of age: A 35-year experience. J. Am. Coll. Surg..

[58] Huang C.-H., Chao T.-C., Hseuh C., Lin K.-J., Ho T.-Y., Lin S.-F., Lin J.-D. (2012). Therapeutic outcome and prognosis in young patients with papillary and follicular thyroid cancer. Pediatr. Surg. Int..

[59] Lamartina L., Leboulleux S., Schlumberger M. (2021). Thyroid cancer incidence in children and adolescents. Lancet Diabetes Endocrinol..

[60] Hogan A.R., Zhuge Y., Perez E.A., Koniaris L.G., Lew J.I., Sola J.E. (2009). Pediatric thyroid carcinoma: Incidence and outcomes in 1753 patients. J. Surg. Res..

[61] Borson-Chazot F., Causeret S., Lifante J.-C., Augros M., Berger N., Peix J.-L. (2004). Predictive factors for recurrence from a series of 74 children and adolescents with differentiated thyroid cancer. World J. Surg..

[62] Hay I.D., Johnson T.R., Kaggal S., Reinalda M.S., Iniguez-Ariza N.M., Grant C.S., Pittock S.T., Thompson G.B. (2018). Papillary thyroid carcinoma (PTC) in children and adults: Comparison of initial presentation and long-term postoperative outcome in 4432 patients consecutively treated at the Mayo Clinic during eight decades (1936–2015). World J. Surg..

[63] Alzahrani A.S., Alswailem M., Moria Y., Almutairi R., Alotaibi M., Murugan A.K., Qasem E., Alghamdi B., Al-Hindi H. (2019). Lung metastasis in pediatric thyroid cancer: Radiological pattern, molecular genetics, response to therapy, and outcome. J. Clin. Endocrinol. Metab..

[64] Chesover A.D., Vali R., Hemmati S.H., Wasserman J.D. (2021). Lung metastasis in children with differentiated thyroid cancer: Factors associated with diagnosis and outcomes of therapy. Thyroid.

[65] Nies M., Vassilopoulou-Sellin R., Bassett R.L., Yedururi S., Zafereo M.E., Cabanillas M.E., Sherman S.I., Links T.P., Waguespack S.G. (2021). Distant metastases from childhood differentiated thyroid carcinoma: Clinical course and mutational landscape. J. Clin. Endocrinol. Metab..

[66] Cistaro A., Quartuccio N., Garganese M.C., Villani M.F., Altini C., Pizzoferro M., Piccardo A., Cabria M., Massollo M., Maghnie M. (2022). Prognostic factors in children and adolescents with differentiated thyroid carcinoma treated with total thyroidectomy and RAI: A real-life multicentric study. Eur. J. Nucl. Med. Mol. Imaging.

[67] Redlich A., Luster M., Lorenz K., Lessel L., Rohrer T.R., Schmid K.W., Frühwald M.C., Vorwerk P., Kuhlen M. (2022). Age, American Thyroid Association risk group, and response to therapy are prognostic factors in children with differentiated thyroid cancer. J. Clin. Endocrinol. Metab..

[68] Ceccarelli C., Pacini F., Lippi F., Elisei R., Arganini M., Miccoli P., Pinchera A. (1988). Thyroid cancer in children and adolescents. Surgery.

[69] Hallwirth U., Flores J., Kaserer K., Niederle B. (1999). Differentiated thyroid cancer in children and adolescents: The importance of adequate surgery and review of literature. Eur. J. Pediatr. Surg..

[70] Dottorini M.E., Vignati A., Mazzucchelli L., Lomuscio G., Colombo L. (1997). Differentiated thyroid carcinoma in children and adolescents: A 37-year experience in 85 patients. J. Nucl. Med..

[71] Lebbink C.A., Links T.P., Czarniecka A., Dias R.P., Elisei R., Izatt L., Krude H., Lorenz K., Luster M., Newbold K. (2022). 2022 European Thyroid Association Guidelines for the management of pediatric thyroid nodules and differentiated thyroid carcinoma. Eur. Thyroid J..

[72] Sharma R.K., Patel S., Gallant J.-N., Esianor B.I., Duffus S., Wang H., Weiss V.L., Belcher R.H. (2022). Racial, ethnic, and socioeconomic disparities in the presentation and management of pediatric thyroid cancer. Int. J. Pediatr. Otorhinolaryngol..

[73] Zhao H.H., Wilhelm S.M. (2023). Pediatric thyroid cancer: Socioeconomic disparities and their impact on access to care. Surgery.

